# Characterizing *Kaempferia parviflora* extracellular vesicles, a nanomedicine candidate

**DOI:** 10.1371/journal.pone.0262884

**Published:** 2022-01-25

**Authors:** Variya Nemidkanam, Nuntaree Chaichanawongsaroj

**Affiliations:** 1 Department of Clinical Chemistry, Graduate Program in Clinical Biochemistry and Molecular Medicine, Faculty of Allied Health Sciences, Chulalongkorn University, Bangkok, Thailand; 2 Department of Transfusion Medicine and Clinical Microbiology, Research Unit of Innovative Diagnosis of Antimicrobial Resistance, Faculty of Allied Health Sciences, Chulalongkorn University, Bangkok, Thailand; Central University of Rajasthan, INDIA

## Abstract

Plant-derived extracellular vesicles (EVs) are a promising candidate for nanomedicine delivery due to their bioactive cargos, high biocompatibility to human cells, biodegradability, low cytotoxicity, and potential for large-scale production. However, the research on EVs derived from medicinal plants is very limited. In this study, *Kaempferia parviflora* extracellular vesicles (KPEVs) were isolated by differential and sucrose density gradient centrifugation, and their size, morphology, and surface charge were characterized using transmission electron microscopy and dynamic light scattering. The biological properties of KPEVs, including their bioactive compound composition, gastric uptake, cytotoxicity, acid tolerance, and storage stability, were also examined. In addition, KPEVs had an average and uniform size of 200–300 nm and a negative surface charge of 14.7 ± 3.61 mV. Moreover, 5,7-dimethoxyflavone, the major bioactive compound of KP, was packaged into KPEVs. Meanwhile, KPEVs were resistant to gastric digestion and stably maintained at −20°C and −80°C for 8 weeks with no freeze-thaw cycle. The lipid hydrolysis during EVs storage at room temperature and 4°C were also demonstrated for the first time. Furthermore, the labeled KPEVs were internalized into adenocarcinoma gastric cells, and the cell viability was reduced in a dose-dependent manner, according to the results of the thiazolyl blue tetrazolium assay. Our study supports the potential application of KPEVs as a vehicle for anticancer or oral drugs.

## Introduction

Oral drug administration is the main route for administering drugs for various infectious and noninfectious diseases due to its noninvasiveness, convenience, and high patient compliance. However, drug bioavailability depends on many factors, such as stability in a highly acid environment (pH 1.2–2.2), the solubility of a drug in the gastrointestinal tract, absorption efficiency, and permeability through the mucus membrane barrier [1]. Thus, there is a need to identify novel strategies that enable more effective oral drug delivery.

In recent years, it has been found that plant-derived extracellular vesicles (PDEVs) can potentially help overcome such limitations. PDEV biogenesis naturally occurs *via* the blebbing, budding, and shedding of plasma membranes or exocytosis of multivesicular bodies [2]. Thus, the membrane composition of PDEVs resembles that of living cells. In addition, biomolecules in the cellular cytoplasm, including proteins, lipids, and nucleic acids, are assembled inside the lumen of extracellular vesicles (EVs) [3].

Moreover, PDEVs offer health benefits. For example, sulforaphane-containing EVs from broccoli can prevent intestinal inflammation by activating dendritic cell AMP-activated protein kinase [4]. In addition, grape EVs can exert a protective and renewing effect on intestinal cells [5]. Moreover, the anticancer activity of lemon EVs enhances cancer cell apoptosis, specifically suppressing chronic myeloid leukemia *in vitro* and *in vivo* [6]. Furthermore, a miRNA profiling study identified 11 edible fruits and vegetables associated with human inflammatory responses and cancer-related genes [7]. Therefore, PDEVs are highly biocompatible with human cells; they may help transfer biomolecules into human cells to exert their biological activities [3]. Additionally, PDEVs have physical features that can enhance drug delivery. For example, PDEVs are 50–500 nm nanoparticles. Moreover, PDEVs have a spherical ultrastructure and high dispersion rates. In addition to low cytotoxicity, these features make PDEVs more promising candidate vehicles for drug delivery, especially *via* the oral route, than other synthetic nanoparticle-based therapy [8].

Therefore, PDEVs represent a novel therapeutic strategy acting as nanodelivery vehicles. For example, grape- and grapefruit-derived EVs can migrate through the intestinal barrier and be absorbed by intestinal stem cells in mice. In addition, the results of treatment with these PDEVs showed a lower mortality rate and prevention of dextran sulfate sodium (DSS)-induced colitis progression in mice [5, 9]. Moreover, intestinal epithelial cells and macrophages internalize ginger EVs; they can modulate inflammatory cytokine response and prevent colitis-associated cancer in a mouse colitis model [10].

While most PDEVs studies have been documented in fruit and vegetable sources, such as ginger, grape, grapefruit, and broccoli [11], only a few studies have been conducted with medicinal plant EVs. Thus, there is a need to further explore the pharmacological applications of medicinal plant EVs. For example, *Kaempferia parviflora* (KP) has been used in traditional medicine since ancient times. A crude KP extract exerts various biological and pharmacological effects, including antiinflammatory, antioxidant, anticancer, and antibacterial activities [12–15]. The KP is effective as a gastrointestinal tract treatment in reducing the size of gastric lesions and enhancing mucosal protection in a mouse model [16]. In our previous studies, we showed that KP methanol extract exhibited superior inhibitory growth activity (MIC = 64 μg/ml) against *H*. *pylori*, the causative agent of gastric cancer. Moreover, KP extracts also exerted anti-internalization activity on *H*. *pylori* in Hep-2 cells [17, 18] and antiinflammatory effect by regulating proinflammatory cytokine expression and leukocyte chemotaxis in *H*. *pylori-*infected gastric cells [19]. Lastly, many flavonoids, such as 5,7-dimethoxyflavone (DMF), 5,7,4-trimethoxyflavone (TMF), and 3,5,7,4-tetramethoxyflavone, have been identified in KP root extracts, which serve as a source of therapeutically active compounds [20].

This study aimed to characterize the physical and biochemical properties and bioactive compounds of KPEVs. In addition, the factors affecting the potential application of KPEVs for oral drug delivery, including stability in acidic pH, cellular uptake, and cytotoxicity, were examined in gastric cells. The insights from this study will be useful for applying KPEVs in drug encapsulation for therapeutic purposes.

## Materials and methods

### Human gastric cell lines

Adenocarcinoma gastric AGS (CRL-1739^™^) (ATCC, VA, USA) were cultured in RPMI 1640 supplement with 10% (v/v) fetal bovine serum (Hyclone, UT, USA) at 37°C in 5% CO_2_ and 80% humidity. After the cells reached 80%–90% confluency, they were harvested using 0.25% EDTA-trypsin (Caisson Labs, UT, USA) and subcultured.

### Isolation and purification of KPEVs

*K*. *parviflora* (KP) rhizomes were purchased from a local farm in Khao Kho, Phetchabun, Thailand (voucher number ES280306). The KPEVs were isolated according to the method of Sung et al. [21] with modifications. First, the KP rhizomes were thoroughly washed with tap water and grounded in a whole slow juicer (B1700, Kuvings). Next, the KP juice was centrifuged sequentially at 1,000 × g for 10 min, 2,000 × g for 20 min, 4,000 × g for 30 min, and 10,000 × g for 60 min (Sorvall^™^ Legend^™^ XT/XF Centrifuge, Fiberlite^™^ F14-6x250 rotor) to remove fiber and cell debris. The resultant supernatant was then pelleted by ultracentrifugation at 150,000 × g for 2 h (Sorvall^™^ WX100+ Ultracentrifuge, T647.5 rotor, K_adj_ = 201). Afterward, sucrose gradient centrifugation (8%/30%/45% sucrose) was performed at 150,000 × g for 1.5 h at 4°C (Hitachi Koki Himac CS150NX, S55A rotor, K_adj_ = 73.4). Finally, the KPEV pellets were resuspended in 1X PBS (GE Healthcare, IL, USA) through mild ultrasonic dispersion, pooled, filtered with a 0.45-μm filter, and stored at −80°C until use.

### Protein quantitation

Total protein content was measured and used to indicate the amount of KPEVs. Protein concentration (μg/ml) was determined using the Bradford method with a Bio-Rad protein quantification assay kit (Bio-Rad, CA, USA) and measured the absorbance at 595 nm using a microplate reader (BioTek, VT, USA). The KPEV samples and bovine serum albumin (BSA) standards were measured at least in triplicates. The concentration of the KPEV protein was calculated using the BSA standard curve. The final yield was calculated and reported as KPEV total proteins per fresh KP rhizome weight (mg/kg).

### Negative staining and transmission electron microscopy (TEM)

The morphology of the KPEVs was observed using TEM. Ten microliters of KPEVs were placed on a carbon-coated copper grid and incubated for 2 min. Then, the KPEVs were negatively stained with 1% uranyl acetate for 3 min. After the grid was air-dried for 10 min, it was imaged using a JEM-1400 electron microscope (JEOL, Japan) at an accelerating voltage of 100 kV.

### Size distribution and surface charge

The KPEVs were diluted in 1X PBS buffer and analyzed using dynamic light scattering with a Zetasizer Nano ZS (Malvern, UK) and a He–Ne laser light source at 633 nm at 25°C. The scattered light was detected at 173° and 13° angles to measure dynamic light scattering (size) and electrophoretic mobility (surface charge), respectively. Each sample was measured 3 times with 30 runs per measurement.

### Identification of active compound cargos in KPEVs

The KPEVs were lysed, dissolved in 100% methanol at 4°C overnight, and subsequently identified and quantified under conditions according to those reported by Malakul et al. [22]. Briefly, the lysed sample was centrifuged at 7,000 × g for 10 min. The DMF standard and samples (Sigma-Aldrich, MO, USA) were examined using HPLC (Shimadzu, Japan). The analytes were separated on 5-μm Purospher^®^ reverse phase column C18 columns (4.0 × 250 mm) (Merck Millipore, MA, USA), with a methanol:water (70:30 v/v) buffer used as the mobile phase at 40°C and a flow rate of 1.0 ml/min. The DMF was detected by UV-VIS at 210 nm. The HPLC fingerprint of KPEVs lysate was compared with that of the standard DMF and quantified by plotting peak areas against DMF standard concentrations.

### Storage stability of KPEVs

The shelf life and storage temperature of the KPEVs were assessed with different storage durations at 0, 1, 2, 4, and 8 weeks and different temperatures at 4°C, −20°C, and −80°C. Then, the physical properties, including size and zeta potential, of the KPEVs were examined. In addition, lipid hydrolysis in the PDEV membranes was investigated by measuring free fatty acid content, octanoate (C-8) and longer FFA, with a free fatty acid quantification assay fluorometric kit (Abcam, Cambridge, UK). The free fatty acid contents were calculated based on the palmitic acid standards.

The freeze-thaw stability of the KPEVs was examined after freezing them at −80°C and thawing them at room temperature for 1 to 3 cycles. The physical stability of the KPEVs was analyzed by measuring their size and zeta potential.

### *In vitro* digestion assay

KPEVs were incubated in an HCl/pepsin solution, pH 1.2 (Markham, ON, Canada), a simulated gastric buffer, in slow rotation at 95 rpm at 37°C for 1 h [23]. Then, the KPEV size distribution and surface charge were determined using dynamic light scattering with a Zetasizer Nano ZS (Malvern, UK) and KPEV morphology was determined using TEM.

### Uptake of KPEVs by gastric cells

The conditions of KPEV uptake by gastric cells was modified from those reported by Yang et al. [24] and assessed using FM4-64 (Biotium, CA, USA), a lipophilic fluorescence labeling dye. First, the KPEVs at **100** μg/ml were incubated with 3 μg/ml FM4-64 at 4°C in the dark for 1 h. Then, the labeled KPEVs were washed with 1X PBS, transferred to 10-kDa MWCO spin column (PALL, NY, USA), and centrifuged at 10,000 × g for 15 min at 4°C to remove the unbounded FM4-64.

Before labeling, AGS cells were seeded on sterile cover slips placed in a 24-well plate at 8 × 10^4^ cells/well and cultured in a complete medium for 24 h. First, each coverslip was washed with 1X PBS with Ca^2+^, Mg^2+^ (1 mM CaCl_2_, 0.5 mM MgCl_2_) before 10 μg/ml labeled KPEVs or the RPMI 1640 control medium were added to the test and control wells, respectively, and incubated for 6, 12, and 24 h at 37°C. Next, the cells on the coverslips were washed with RPMI 1640, fixed with 1% paraformaldehyde, and nuclear stained with DAPI (Vector Laboratories, CA, USA). The uptake of the labeled KPEVs by the gastric cells was visualized using a confocal laser scanning microscope (Zeiss, Germany) at 515/640 nm (FM 46–4) and 358/461 nm (DAPI). In addition, the localization of the KPEVs in the cells was observed by 2D imaging in the z-stack mode.

### Cytotoxicity of KPEVs

AGS cells were seeded in a 96-well plate at 1 × 10^4^ cells/well and incubated in a complete medium (RPMI with 10% FBS) without antibiotics for 24 h. The cells were then washed with 1X PBS with Ca^2+^, Mg^2+^ (1 mM CaCl_2_, 0.5 mM MgCl_2_) and incubated at 37°C with 5, 10, 30, 50, or 100 μg/ml KPEVs for 24 h. Then, 5 mg/ml thiazolyl blue tetrazolium bromide (MTT) was added to each well for a 4-h incubation at 37°C in the dark. Next, the cells were permeabilized in 100 μl of 10% (w/v) SDS overnight to solubilize the intracellular formazan crystals. Lastly, the absorbance of the cells was read at 570 nm, and the percentage of viable cells was calculated compared to the untreated control cells.

### Statistical analysis

The experiments were performed in at least triplicates, and the data are presented as a mean ± standard deviation (SD). Graph plotting and statistical analysis were performed using GraphPad Prism 9 (GraphPad Software, San Diego, CA, USA.). The difference between the control and test groups was analyzed using one-way analysis of variance (ANOVA) followed by the Bonferroni test. The effects of the storage temperatures on the KPEVs over time were analyzed using two-way ANOVA followed by the Bonferroni test. In addition, p-values of less than 0.05 were considered statistically significant.

## Results

### Isolation and characterization of KPEVs

**Fig 1:**
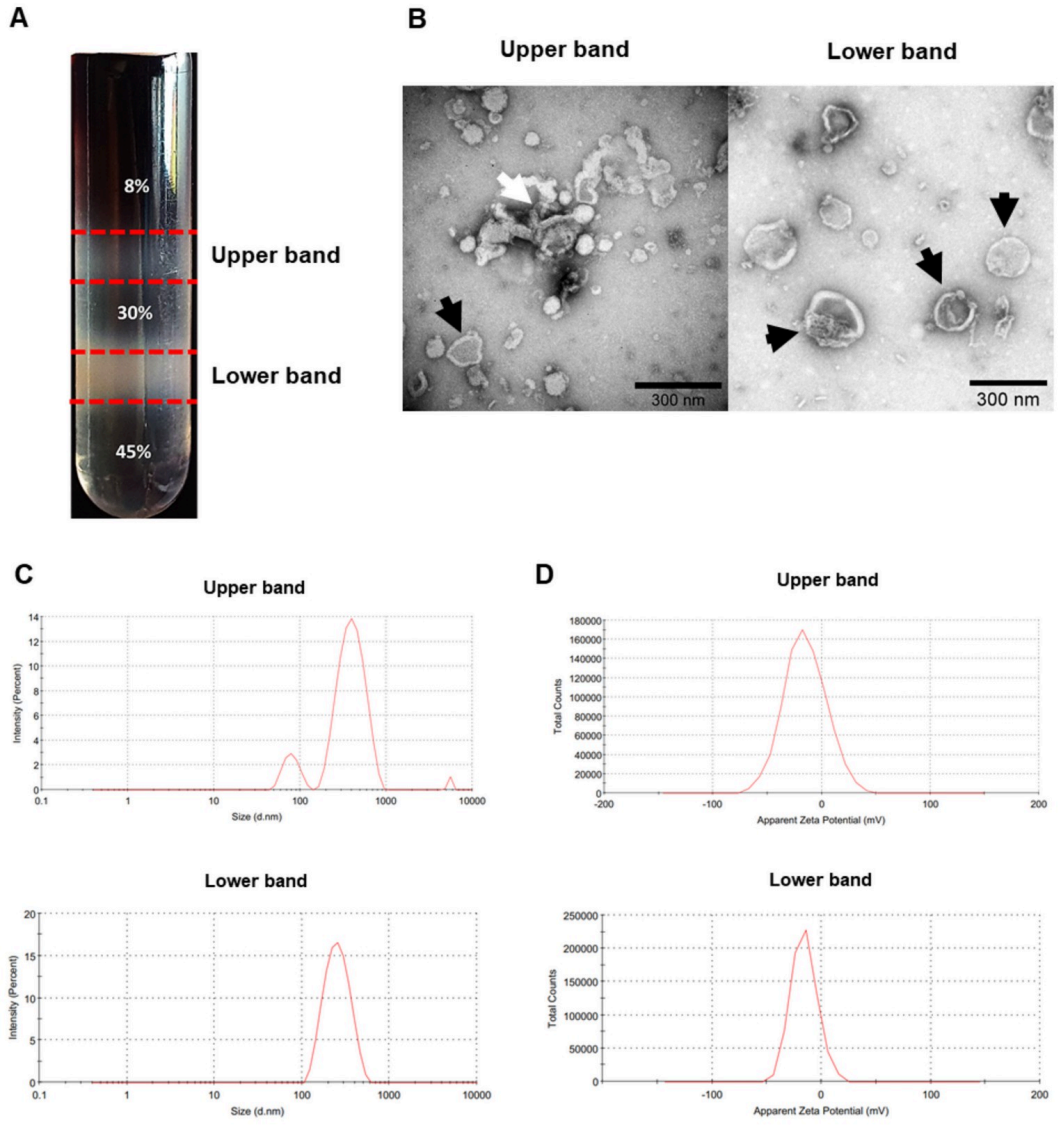
Characterization of KPEVs. (A) Upper and lower purified KPEVs population in sucrose density gradient after ultracentrifugation. (B) The morphology of each band of KPEVs was visualized by TEM; the scale bar indicates 300 nm. (C) The size distribution and (D) zeta potential of the KPEVs were determined by DLS using a Zetasizer Nano ZS.

The KPEVs were isolated from KP juice by ultracentrifugation and sucrose density gradient. Two EV populations were observed (Fig 1A). Most EVs were in the lower band, or in the 30% to 45% part of the sucrose gradient. According to the TEM imaging, a homogeneous population of cup-shaped 200–300 nm EVs could be observed in the lower band (Fig 1B, Black arrows). Meanwhile, an upper band of the EV population was also observed between the 8% to 30% part of the sucrose gradient, likely with cell debris aggregation (Fig 1B, White arrow).

Corresponding to the TEM imaging data, the dynamic light scattering data showed the average size of the KPEVs was 256.8 ± 14.81 and 346.8 ± 37.43 nm was for the lower and upper bands, respectively (Fig 1C). In addition, a polydispersity index (PDI), representing a scale of size heterogeneity of the particles, was measured to be 0.252 ± 0.055 and 0.407 ± 0.086 for the lower and upper bands, respectively. Moreover, the negative surface charge was −14.7 ± 3.61 and −16.1 ± 4.94 mV for the lower and upper bands, respectively (Fig 1D). Lastly, the final yield of the KPEVs from fresh KP rhizome was about 3.6 mg/kg. Therefore, only the KPEVs in the lower band were collected for further analysis.

### An active medicinal compound incorporated in the KPEVs

**Fig 2:**
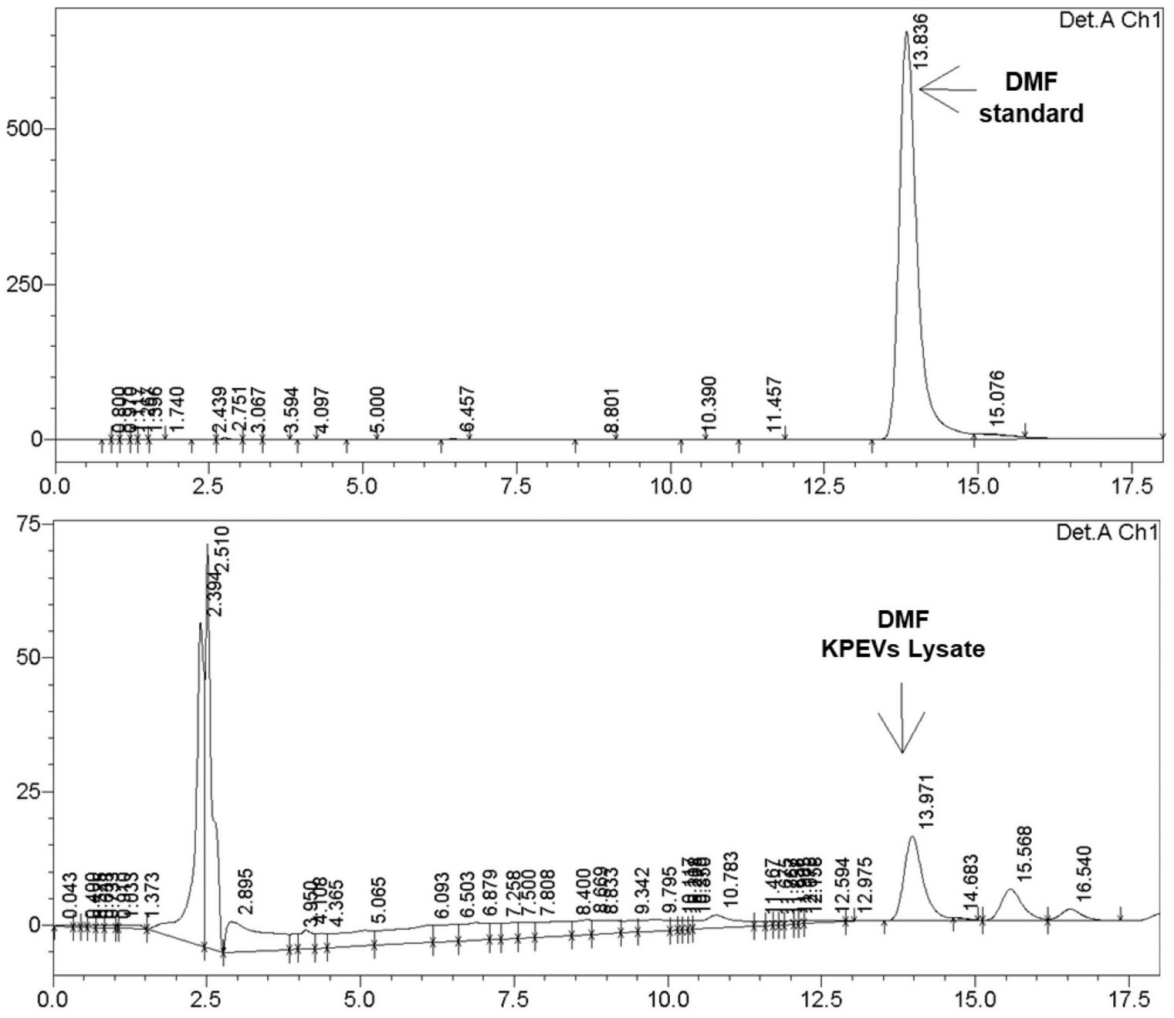
Qualification of 5,7-Dimethoxyflavone (DMF) content in KPEVs using HPLC. The DMF in KPEVs lysate samples was identified by comparison to the DMF reference standard and quantified by the DMF standard curve.

As the quantity of bioactive compounds in KPEV nanoparticles is significantly lower than that of KP crude extracts, only 5,7-dimethoxyflavone (DMF) the most crucial KP rhizome active medicinal compound, was identified and quantified compared to a reference standard. According to the HPLC results (Fig 2), the KPEV lysate contained DMF; the peak of DMF in the lysate was eluted at the retention time of 13.97 min, similar to the retention time of standard DMF at 13.83 min. In addition, the concentration of DMF in the KPEV lysate was 13.32 ± 5.40 ng in 1 μg of KPEV protein.

### Storage temperature stability of KPEVs

**Fig 3:**
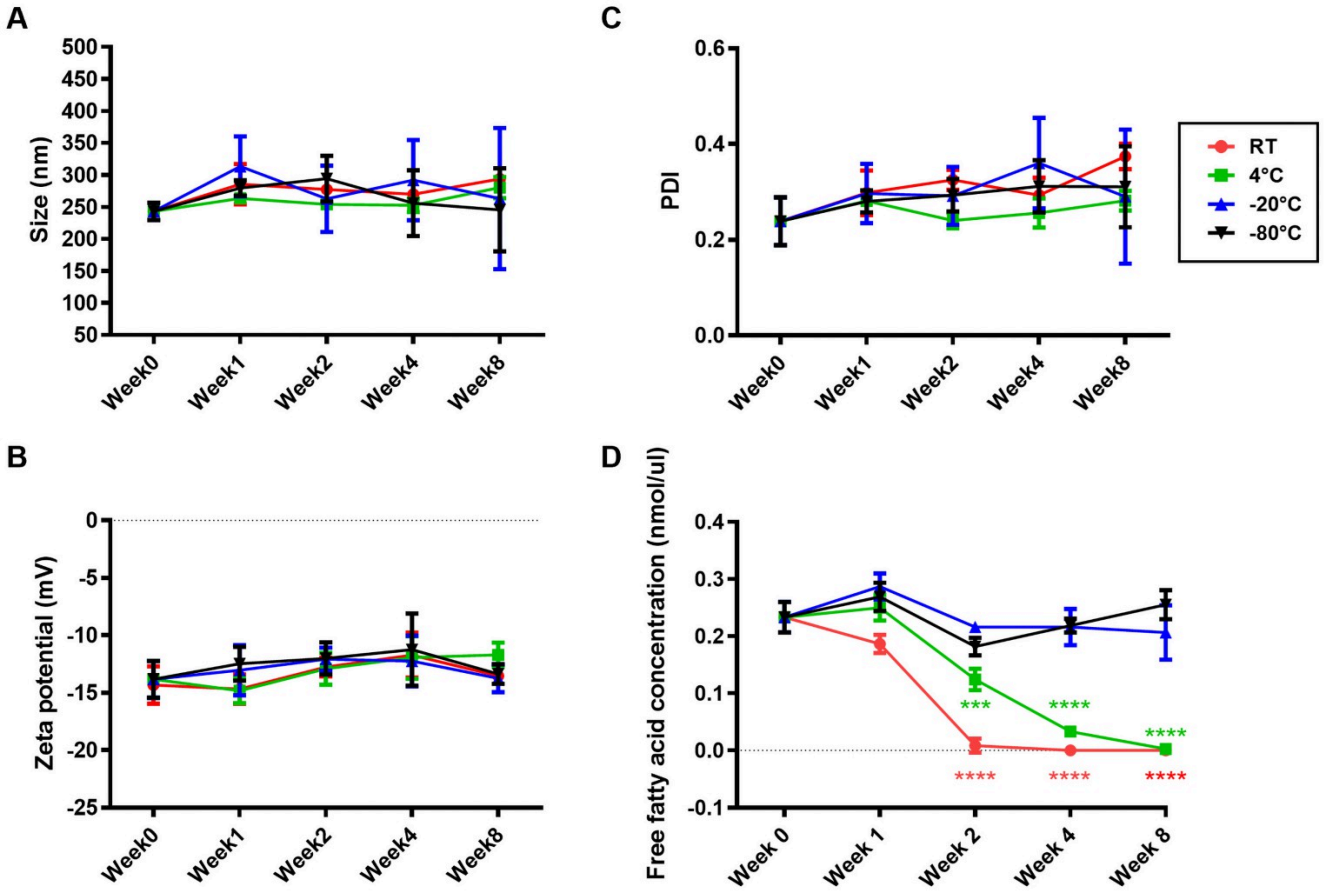
Determination of KPEVs stability in different storage temperatures. The (A) size distribution, (B) zeta potential, and (C) PDI of the KPEVs stored at 4°C, −20°C, or −80°C for 0, 1, 2, 4, or 8 weeks were analyzed by DLS. (D) The free fatty acid contents of the stored KPEVs were analyzed using an ELISA kit. The experiments were performed in at least triplicates, and the data are presented as the mean ± SD. The statistical analysis was performed using two-way ANOVA followed by the Bonferroni test. (***) and (****) indicate P-values of *p* ≤ 0.001 and *p* ≤ 0.0001 compared to KPEVs on week 0, respectively.

**Fig 4:**
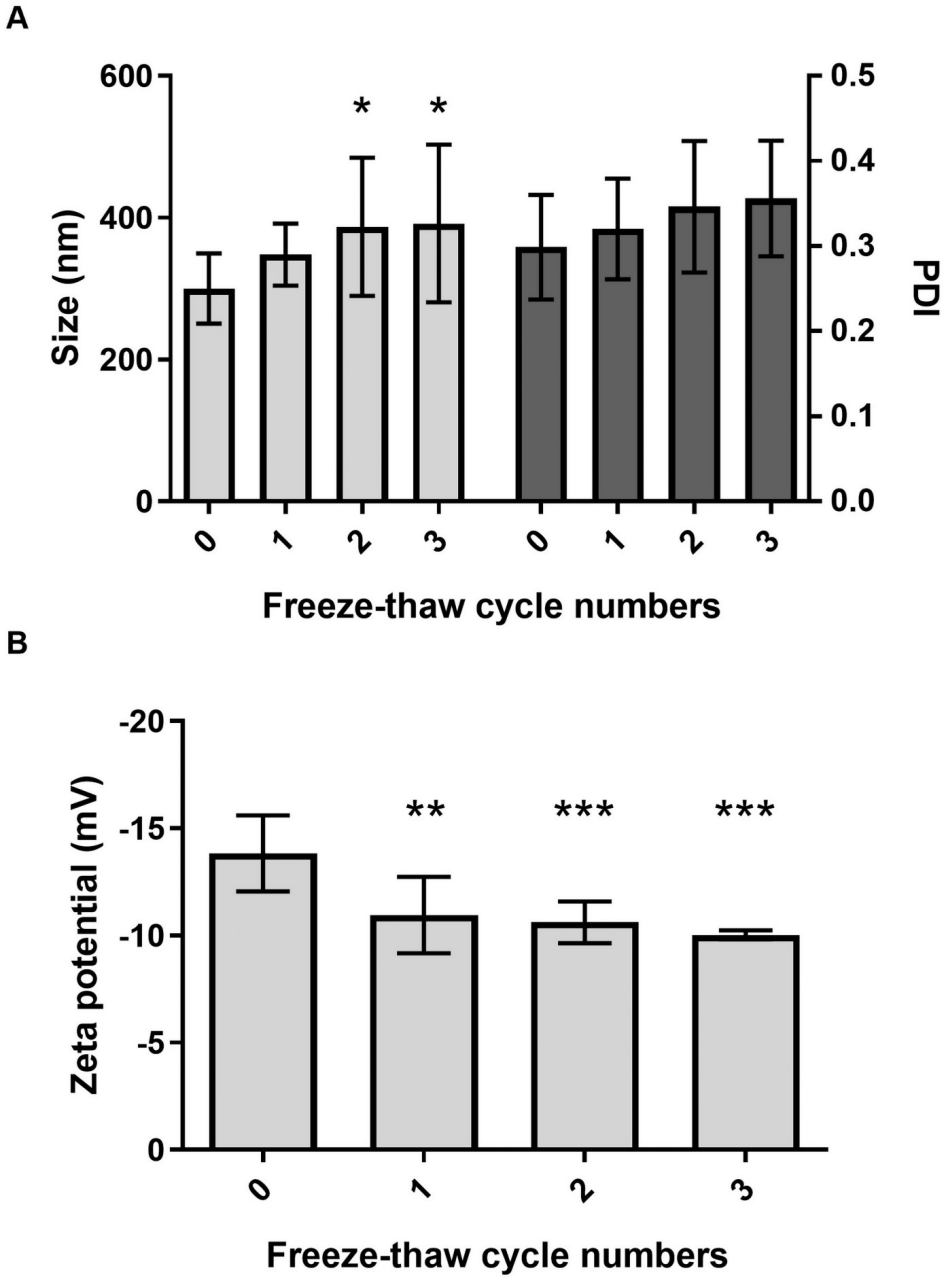
Determination of KPEVs stability after freeze-thaw cycles. The size distribution (A, left), PDI (A, right), and zeta potential (B) of the KPEVs after 0 to 3 cycles were analyzed using DLS. The experiments were performed in at least triplicates, and the data are presented as the mean ± SD. The statistical analysis was performed using two-way ANOVA followed by the Bonferroni test. (*), (**), and (***) indicate P-values of *p* ≤ 0.05, *p* ≤ 0.01, and *p* ≤ 0.001 compared to freeze-thaw cycle 0.

The size, PDI, and zeta potential of the KPEVs were not significantly changed until after 8 weeks of storage at 4°C, −20°C, and −80°C (Fig 3A, 3B and 3C). PDI of the KPEVs was gradually increased over time from week 1 to 4 at room temperature (RT) (Fig 3C). Interestingly, the free fatty acids, the main component in the KPEV membrane, were rapidly degraded since week 1 and completely depleted in week 2 at RT. Although the loss of free fatty acid at 4°C was slower than at RT, the KPEVs were completely degraded at week 8. In contrast, the free fatty acid content was maintained under frozen conditions at −20°C and −80°C from 0 to 8 weeks. Moreover, the stability of the KPEVs was reduced by more than 1 freeze-thaw cycle (Fig 4A, left). The size of the KPEVs was significantly increased from 300.3 ± 49.65 to 387.1 ± 97.53 nm and 391.9 ± 111.1 nm in the second and third freeze-thaw cycles, respectively. Although the PDI value of the KPEVs did not significantly change in repeated freeze-thaw cycles, the heterogeneity of the KPEVs had an increasing trend (Fig 4A, right). Lastly, the negative charge of the KPEVs was reduced from the first to third freeze-thaw cycle, as the zeta potential shifted from −13.83 ± 1.778 to −10.96 ± 1.787 from the first to the second cycle and from −10.62 ± 0.9672 to −10.03 ± 0.2095 from the second to the third cycle (Fig 4B).

### Tolerance of KPEVs under a stomach-like condition

**Fig 5:**
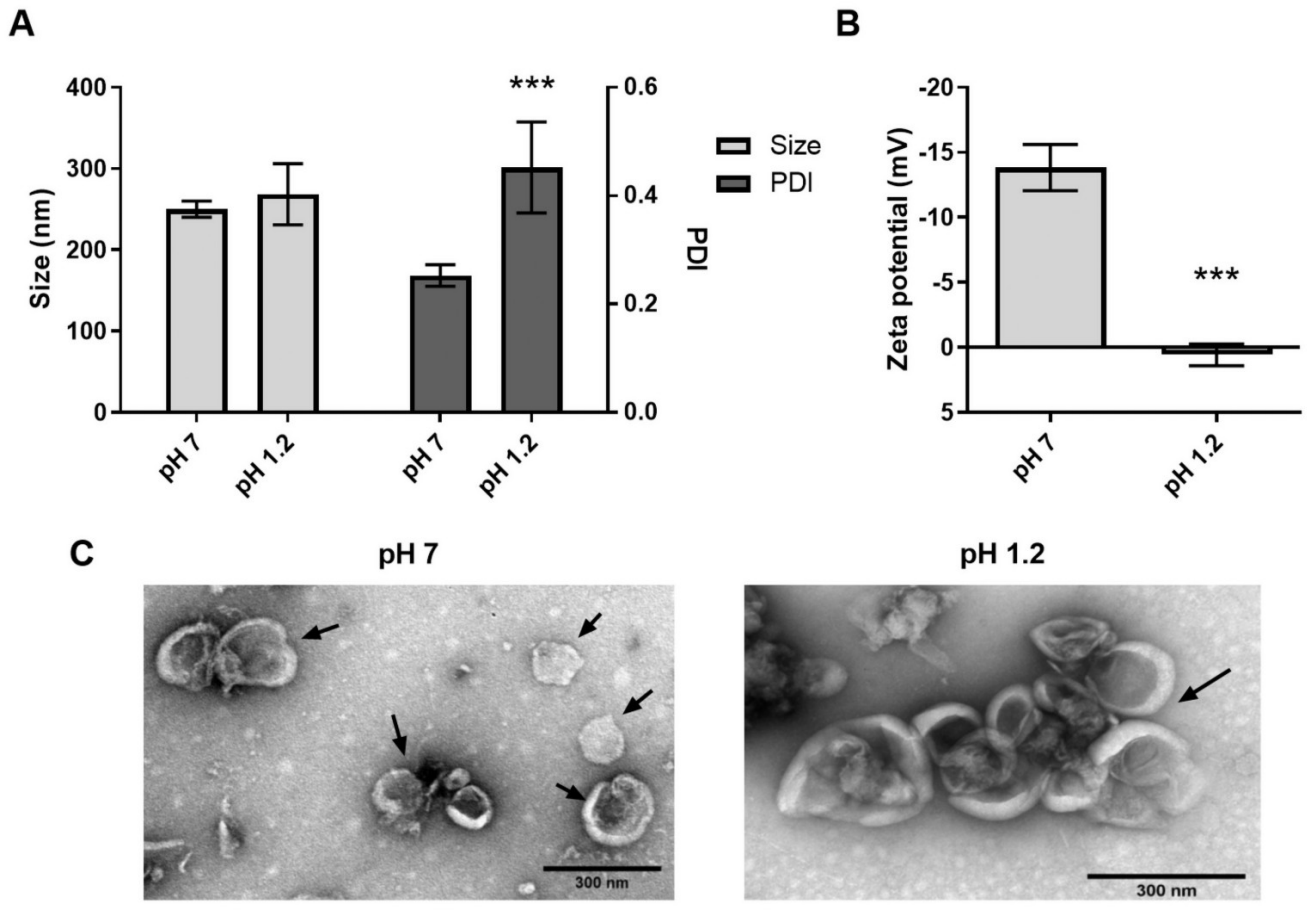
The tolerance of the KPEVs under a simulated gastric condition. The size distribution (A, left), PDI (A, right), and zeta potential (B) of the KPEVs before and after incubation under the simulated gastric condition. (C) KPEV morphology in neutral pH 7.0 and acid pH 1.2 were visualized by TEM; the scale bar indicates 300 nm. The experiments were performed in at least triplicates, and the data are presented as the mean ± SD. The statistical analysis was performed using one-way ANOVA followed by the Bonferroni test. (***) indicates a P-value of *p* ≤ 0.001 compared to KPEVs at pH 7.0.

The KPEV acid resistance mimics stomach conditions, assessed at 37°C for 1 h by suspending and rotating the KPEVs in a stimulated gastric buffer, pH 1.2 compared to 1X PBS, pH 7.0. After acid digestion, the size of the KPEVs slightly enlarged as their diameter shifted from 250.00 ± 10.00 nm (pH 7.0) to 268.40 ± 37.73 nm (pH 1.2), and their PDI value increased from 0.25 ± 0.02 (pH 7.0) to 0.45 ± 0.08 (pH 1.2) (Fig 5A). In addition, the surface charge of the KPEVs shifted from −15.7 ± 1.2 (pH 7.0) to 0.57 ± 0.8 mV (pH 1.2) (Fig 5B). The TEM image supported the DLS results, the digested KPEV morphology remained intact, slightly increasing in size (230–320 nm) and clumping together at pH 1.2 compared to KPEVs at pH 7.0 (Fig 5C, Black arrows).

### The uptake of KPEVs by human gastric cells

**Fig 6:**
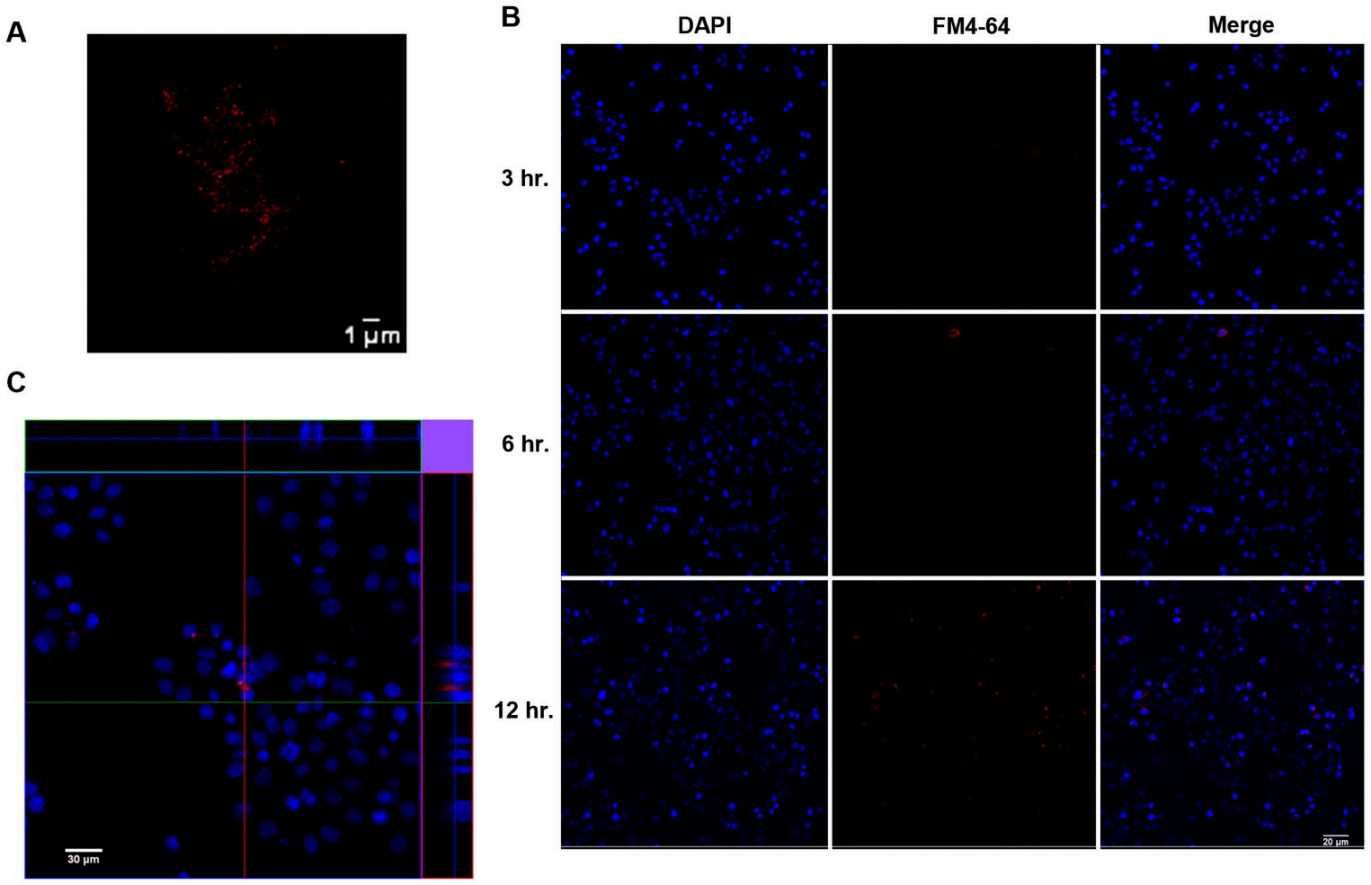
Confocal microscope images of the uptake of KPEVs in human gastric cells. (A) KPEVs were labeled with FM4-64 (red), a lipophilic dye. (B) AGS cells were cocultured with the labeled KPEVs for 3, 6, and 12 h. The cell nuclei were stained with DAPI (blue). (C) The ortho view of the Z-stack images was taken at 12 h.

FM4-64, a lipophilic fluorescence dye that stained KPEV membranes red (Fig 6A), was used to track the gastric uptake of KPEVs using a confocal laser scanning microscope. After AGS cells were coincubated with FM4-64-labeled KPEVs, a gradual increase of KPEV uptake was observed over time at 3, 6, and 12 h (Fig 6B). In addition, the labeled KPEVs were internalized into the AGS cells and localized in the cytoplasm after coincubation for 12 h; the labeled KPEVs could be seen around the DAPI-stained nucleus (blue), according to the Z-stack 3D imaging results (Fig 6C).

### KPEVs induced cytotoxicity in gastric cancer cells

**Fig 7:**
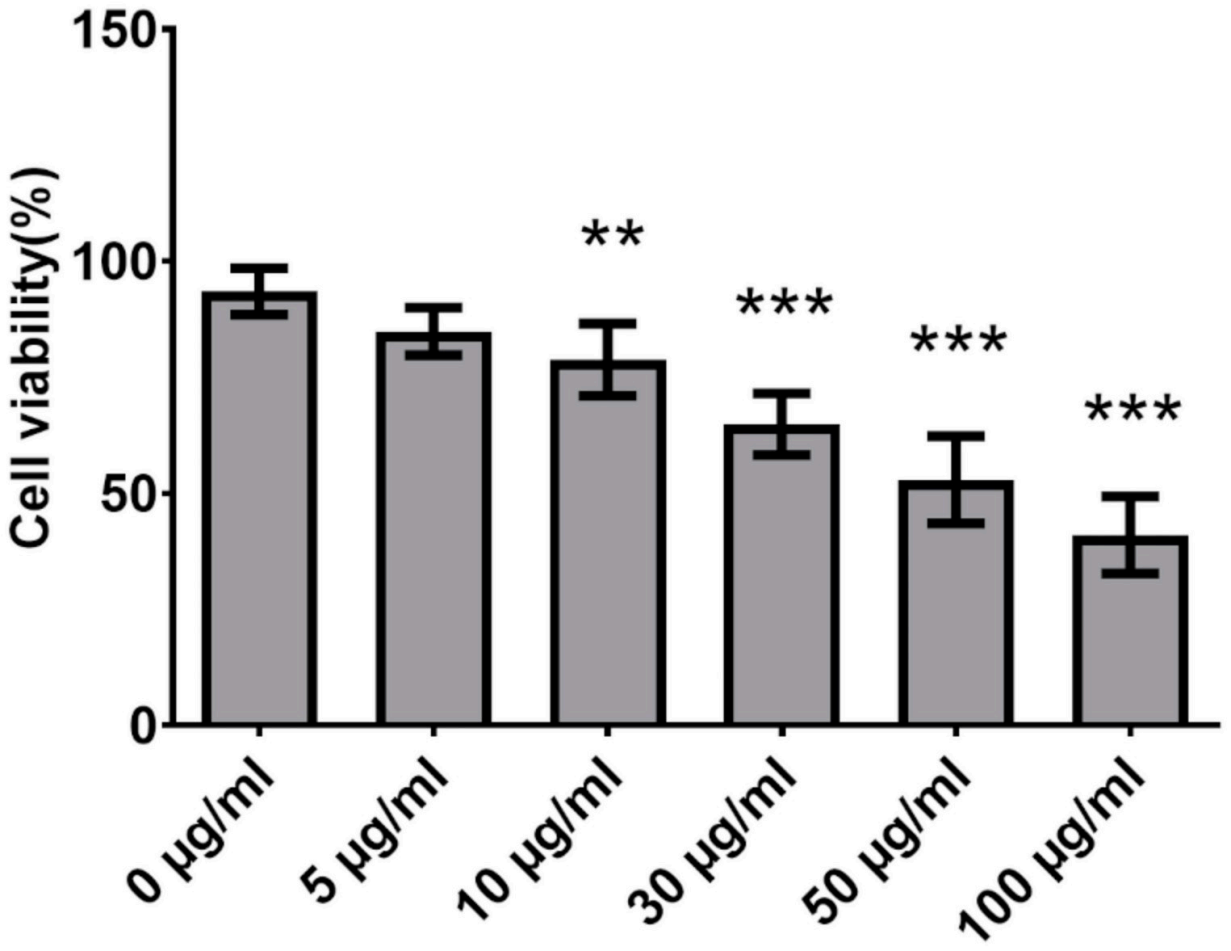
Cytotoxic effect of KPEVs on gastric cancer cells. The viability of the AGS cells was determined using the MTT assay after incubated with various concentrations of KPEVs for 24 h. The experiments were performed in at least triplicates, and the data are presented as the mean ± SD. The statistical analysis was performed using one-way ANOVA followed by the Bonferroni test. (**) and (***) indicate a P-value of *p* ≤ 0.01 and *p* ≤ 0.001 compared to the media control.

The cytotoxicity of the KPEVs to the gastric cancer cells was examined by coculturing AGS cells with various concentrations of KPEVs for 24 h. At the lowest concentration of 5 μg/ml, the KPEVs did not affect cell viability (84.90% ± 5.10%). In contrast, higher KPEV concentrations at 10, 30, 50, and 100 μg/ml significantly decreased cells viability to 78.76% ± 7.76%, 64.84% ± 6.66%, 52.90% ± 9.41%, and 41.00% ± 8.30%, respectively (Fig 7). Therefore, KPEVs demonstrated cytotoxicity in a concentration-dependent manner with IC_50_ of 68.29 ± 11.44 μg/ml.

## Discussion

Plant-derived extracellular vesicles have promising therapeutic applications, especially in drug nanodelivery systems [25]. EVs from medicinal plants are interesting due to their pharmacological properties, low cytotoxicity, and extensive use since ancient times. However, few medicinal plants have been isolated, characterized, and examined for their biological properties.

*Kaempferia parviflora* (KP) rhizome, or black ginger, is a traditional remedy for gastrointestinal disorders. KP potions are prepared by decoction and consumed to relieve excessive flatulence and gastric ulcer [26]. KP rhizomes have many pharmacological activities, including anticancer, antiinflammatory, antioxidant, and antibacterial [12–15, 27]. In this study, KPEVs were isolated using the differential and density gradient ultracentrifugation, the most common method for isolating PDEVs [11, 28]. This method represents a discontinuous gradient and used less centrifugation time than isopycnic centrifugation [29, 30] and achieves a high purity and good separation of EV subpopulations from smaller, soluble molecules and contaminants in different buoyant densities, effectively maintaining the EV membrane integrity and biological properties. However, this method is disadvantageous because it is time-consuming, cumbersome, and challenged by yield loss from multiple centrifugation steps [31].

Here, the yield of our KPEVs was higher than the yield from animal samples and mammalian cells culture medium [32]. In addition, the type, quantity, and quality of PDEVs likely depend on many factors, including the part or organ of a plant, such as fruit, leaf, seed, stem, and root, and isolation techniques. For example, 250 ml of strawberry juice yields approximately 18 mg of nanovesicles [33]. Meanwhile, the entire aerial part of a sunflower provides 1.4–2 μg of EVs per 1 ml of extracellular fluid [34]. Lastly, the amount of lemon vesicle isolated from fruit pulp was 600 μg per 240 ml of juice [6].

In this study, purified KPEVs have a low PDI value, exhibiting an acceptable size homogeneity for lipid-based nanoparticles [35]. In addition, the KPEVs have a typical cup-shaped appearance and negatively charge similar to some PDEVs and mammalian EVs [36–38]. The EVs derived from ginger, a member of *the Zingiberaceae* family, share similar physical properties to our KPEVs [10, 36]. The bioactive compounds included in PDEVs offer therapeutic benefits. For example, gingerol, shogaol, sulforaphane, and ginsenoside have been identified in the EVs derived from ginger, broccoli, and ginseng [4, 10, 39].

Our study was the first to present the analysis of the KPEV cargo, revealing a highest content of 5,7-dimethoxyflavone (DMF), the main methoxyflavone in the KP rhizome [40]. Similarly, the quantification of the ethanolic KP extract yielded 32.88% of 5,7-dimethoxyflavone (DMF), 26% of 5,7,4′-trimethoxyflavone (TMF), 22.01% of 3,5,7,3′,4′-pentamethoxyflavon (PMF), and 2.36% of 5,7,3′,4′-tetramethoxyflavone. The three major compounds significantly suppressed the expression of TNF-α, IL-6, MMP13, and ZIP8 genes [40]. In agreement with Malakul et al, a major component of 5,7-dimethoxyflavone was present in ethanolic KP extract with small amounts of other methoxyflavones including 3,5,7-trimethoxyflavone, 5-hydroxy-7-methoxyflavone, and 5-hydoxy-3, 7-methoxyflavone [22]. Supercritical CO_2_ fluid extracts (SFEs) of KP contained the highest concentration of 5,7-dimethoxyflavone, showing more potent antiproliferative activity against HeLa and human gastric adenocarcinoma AGS cell lines [41]. The amounts and varieties of methoxyflavones depended on the extraction solvents and conditions. A KP rhizome methanolic extract contained 25 flavonoids and three acetophenones with inhibitory effects against melanogenesis [42]. Thirteen methoxyflavones were isolated from several solvents, exhibiting anticholinesterase activity [43]. Other minor bioactive compounds in KPEVs need further characterization. Apart from their internal components, PDEVs can be used for medicinal agent nanodelivery. For example, a common antiinflammatory drug, methotrexate, had been encapsulated in grapefruit EVs, exhibiting therapeutic effects in DSS-induced mouse colitis [5]. Ginger derived exosome-like nanovesicles could deliver siRNA to tumor cells [44]. Therefore, KPEV characteristics are also promising for future use as therapeutic drug nanovectors.

The KP bioactive compounds possessed a remarkable ability to treat gastrointestinal tract disorders, as evidenced by reducing the size of the gastric lesion, enhancing mucosal protection, exerting antiinflammatory effects, and inhibiting *H*. *pylori* infection [16, 18, 19]. Our *in vitro* digestion assay shown that KPEVs were stable under gastric-like conditions as their size was only slightly enlarged and no effect to structural integrity. Under the same condition, the surface charge of the KPEVs was neutralized to a weak positive charge by the hydrogen ions in gastric acid (HCl). Our data is consistent with the finding by Mu et al. that edible plant-derived exosome-like nanoparticles can resist gastric pepsin solutions, exhibiting altered sizes and a remarkable reduction in negative charge in a stomach-mimicking solution [36]. Therefore, KPEVs display a potential for oral nanodrug delivery.

The critical challenge in applying PDEVs is their stability in different storage temperatures, durations, and freeze-thaw cycles. Most plant crude extracts, or EVs are generally kept at −20°C or −80°C, which would stably maintain their biological activities [45]. KPEVs maintained physical stability in size, particle dispersion, and surface charge for a long time at various temperatures. However, lipid degradation occurred at higher temperatures quickly. Long-chain FA (longer than C-8) in membrane phospholipid building blocks may be broken down at temperatures higher than −20°C [46], likely influencing PDEV integrity. Our study is the first to report PDEV lipid hydrolysis.

EV stability has only been explored in mammalian cells. Munagala et al. has reported that bovine milk-derived exosomes are stably maintained at −80°C for up to 6 months and partially degraded at 4°C [47]. Our data suggest that multiple freeze-thaw cycles must be avoided, as shown by enlarged size and reduction in negative charge in KPEVs. In addition, Kumeda et al. showed that the membrane integrity of human saliva-derived exosome decreased during several freeze-thaw cycles at −20°C [48]. Thus, it will be worthwhile to study the stability of PDEVs further for their future application in medical fields.

Although the lipid composition of PDEVs is different from animal and mammalian cells, we confirmed that labeled KPEVs were internalized into gastric cells. The mechanism of PDEV internalization into mammalian cells remains unclear. However, it has been noted that endogenous phosphatidic acid on the PDEVs surface is attributed to the internalization by the endocytosis pathway [49]. Phosphatidic acid acts as a secondary messenger to interact with several proteins and regulate the cell membrane transport system [50, 51]. Besides, the size of PDEVs may influence the mode of endocytosis. After PDEVs are internalized by mammalian cells, they can exert their therapeutic effects, such as the antitumor effect of lemon EVs and antiinflammatory and immune-modulatory activities of ginger EVs [6, 10]. Furthermore, KPEVs exhibited cytotoxicity against AGS cells in a dose-dependent manner similar to our previous study of KP crude extracts [19]. This finding is crucial for selecting a suitable quantity of KPEVs for cancer-killing or drug delivery functions.

## Conclusions

In this study, we demonstrated that KPEVs were nanoparticles enclosed with 5,7-dimethoxyflavone, a major KP bioactive compound. These KPEVs can be internalized into gastric cells and are resistant to gastric acid. In addition, our study is the first to describe the storage stability in PDEVs by documenting the physical characteristics and lipid degradation of our KPEVs. The KPEVs are best maintained until 8 weeks at −20°C and −80°C with a single freeze-thaw cycle. Moreover, the cytotoxic effect of KPEVs is related to their concentrations. Therefore, KPEVs provide an alternative approach for enhancing gastric cancer therapy as a drug delivery vehicle.
